# Concentration Levels and Ecological Risks of Persistent Organic Pollutants in the Surface Sediments of Tianjin Coastal Area, China

**DOI:** 10.1155/2013/417435

**Published:** 2013-01-16

**Authors:** Xiaoxia Lu, Chaoqi Chen, Shu Zhang, Zhen Hou, Junjun Yang

**Affiliations:** Ministry of Education Laboratory for Earth Surface Processes, College of Urban and Environmental Sciences, Peking University, Beijing 100871, China

## Abstract

Sediments were sampled from different surface water bodies in Tianjin coastal area, China, and persistent organic pollutants (POPs) including polycyclic aromatic hydrocarbons (PAHs), organochlorine pesticides (OCPs), polychlorinated biphenyls (PCBs), and polybrominated diphenyl ethers (PBDEs) were measured using GC/MS or GC/ECD. The purposes were to investigate the concentration levels of the POPs and to assess their ecological risks. The results showed that all the 16 priority PAHs were detected from the 10 sediments sampled with the total concentrations of the 16 PAHs ranging from 274.06 **μ**g/kg to 2656.65 **μ**g/kg, while the concentrations of the halogenated POPs were generally low except in the Dagu waste discharging river where the total concentrations of 24 OCPs, 35 PCBs, and 14 PBDEs were 3103.36 **μ**g/kg, 87.31 **μ**g/kg, and 13.88 **μ**g/kg, respectively. In the studied sediments, PAHs exhibited risks to benthonic organisms; particularly the concentrations of naphthalene and/or acenaphthene exceeded their probable effect concentrations in several locations. In comparison, only in the Dagu waste discharging river, OCPs exhibited risks with the concentrations of heptachlor epoxide and lindane exceeding their probable effect concentrations. PCBs and PBDEs posed rare risks in the studied area.

## 1. Introduction

Persistent organic pollutants (POPs) are organic compounds resisting degradation through chemical, biological, and photolytic processes in the environment. They can bioaccumulate through food webs from the environment and pose a risk of causing harmful effects to the ecosystem and human health according to animal experiments and epidemiological studies [1]. Common POPs such as polycyclic aromatic hydrocarbons (PAHs), organochlorine pesticides (OCPs), polychlorinated biphenyls (PCBs), and polybrominated diphenyl ethers (PBDEs) are widely detected in the environment and the organisms [2–10]. Although there are a few natural sources of POPs, most are created by humans in industrial processes (details about the source analysis of PAHs are presented in the supplemental materials available at http://dx.doi.org/10.1155/2013/417435).

Tianjin is located in the west side of Bohai Bay and the center of the circular Bohai Belt. Depending on its rich natural resources and solid industrial base, Tianjin has become one of the most active and potential regions in economic development in China. Particularly, the development and opening up of the Tianjin coastal area has been fit into the National Eleven Five program and development strategy. However, with the development of economy and urbanization, Tianjin, especially the coastal area, faces increasing environmental problems that cause harm to the health of residents and the development of the city. Researches show that the surface soils in Tianjin coastal area have been widely polluted by PAHs, with the mean value of the total concentration of the 16 priority PAHs  (ΣPAH_16_)  being 1148.10 ng/g [11]. In the sediments of the Dagu waste discharging river, the ΣPAH_16_ ranged from 370 ng/g to 5607 ng/g, and the total concentrations of 9 OCPs (ΣOCP_9_) and 13 PBDEs (ΣPBDE_13_) ranged from 325.3 ng/g to 1389.1 ng/g and 0.1 ng/g to 15.4 ng/g, respectively [12]. However, currently little is known about the concentration levels of POPs in other surface water bodies in the Tianjin coastal area.

Sediment-associated POPs are known to exhibit narcotic effects in benthic organisms, and they also have been implicated in the development of tumors in bottom-feeding fish and in the induction malformation, loss of fertility, or immuno deficiency in many organisms [13]. POPs can enter the aquatic food webs and pose a risk to human health via consumption of seafood [14]. It has been reported that consumption of shellfish polluted by PAHs may cause lung cancer in humans [15]. Therefore, analyzing the ecological risk of POPs is crucial for protecting human health and marine environment security. 

In this study, surface sediments were sampled from different surface water bodies in Tianjin coastal area. The concentrations of POPs including PAHs, OCPs, PCBs, and PBDEs were measured and sources of the POPs were analyzed. Based on the toxicity data of benthic organisms, the ecological risks of POPs were assessed. The purposes were to investigate the concentration levels of PAHs, OCPs, PCBs, and PBDEs in the surface sediments of Tianjin coastal area and to determine their ecological risks. 

## 2. Materials and Methods

### 2.1. Sampling of the Sediments

**Figure 1:**
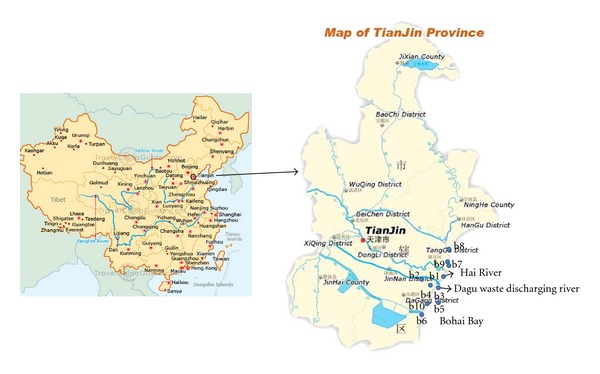
Plot of sediment sampling locations in Tianjin coastal area. b1: mouth of Hai River; b2: Dagu waste discharge river; b3: mouth of Dagu waste discharge river; b4: offshore river; b5: Du waste discharging river; b6: Bei waste discharging river; b7: offshore sea; b8: Yinghe reservoir; b9: mouth of yongding new river; b10: Beitang waste discharging river.

In January 2009, surface sediments were sampled from 10 locations in Tianjin coastal area, as shown in Figure 1. The 10 locations were labeled b1 to b10. Among them, b1, b4, b7, and b9 were normal water bodies, b2, b3, b5, b6, and b10 were waste discharging rivers, and b8 was waste discharging lake. The waste discharging rivers and lake were artificial excavated canals and reservoir that were mainly used for discharging waste water from industry or municipality, while normal water bodies referred to natural water bodies. The locations of two major rivers, Hai River and Dagu waste discharging river, were labeled in Figure 1. A grab dredging device was used to sample the surface sediments. The samples were collected in polyethylene bags with air being squeezed out and transported to the laboratory with ice. After arriving in the laboratory, the samples were stored in a −20°C freezer.

### 2.2. Analysis of Physiochemical Properties of the Sediments

Before analysis, the sediment samples were frozen dried and ground to pass through a 70 mesh metal sieve. Physicochemical properties including pH, available nitrogen (available-N), available phosphate (available-P), and total organic carbon (TOC) of the sediments were analyzed. For the pH, 5 g of each of the dried and sieved samples was thoroughly mixed with 25 mL distilled water and shaken for 3 h in a shaker; the turbid liquid was centrifuged at 5000 rpm for 10 min (*g* value 3773), then the supernatant was filtered through 0.45 *μ*m filter membrane (cellulose acetate) and measured using a pH meter (METTLER DELTA320, Swiss). The available-N was measured using the alkaline hydrolysis diffusion method, and the available-P was measured using the sodium bicarbonate method [16]. The TOC was measured using the TOC analyzer (TOC-5000A, Shimadzu).

### 2.3. Analysis of Persistent Organic Pollutants in the Sediments

Microwave-assisted extraction was applied to extract the POPs from the dried and sieved sediments. For each sample, 5 g of the sediment and 50 ng of each of the surrogate standards (naphthalene-d_8_, acenaphthene-d_10_, anthracene-d_10_, chrysene-d_12_, and perylene-d_12_ for PAHs, 2,4,5,6-tetrachloro-m-xylene for OCPs, C^13^-PCB-209 for PCBs, and C^13^-PCB-141 for PBDEs) were added into a 55 mL extraction vessel of the Microwave Accelerated Reaction System (CEM MARS Xpress, USA) and extracted with 20 mL mixture of n-hexane and acetone (1 : 1, v/v) at 110°C and 1200 W for 20 min. The extract was evaporated to near dryness under reduced pressure at 35°C with a rotary evaporator. The cleanup of the extract was conducted using a multilayer chromatography column (30 cm × 10 mm i.d.). For measuring PAHs and OCPs, a two-layer column (from bottom to top, sequentially packed with aluminium oxide and silica gel) was used; the extract was successively eluted with 20 mL n-hexane and 70 mL dichloromethane. For measuring PCBs, and PBDEs, a five-layer column (from bottom to top, sequentially packed with aluminium oxide, neutral silica gel, alkaline silica gel, neutral silica gel, and acid silica gel) was used; the extract was eluted with 70 mL mixture of n-hexane and dichloromethane (1 : 1, v/v). The eluent was concentrated with the rotary evaporator and transferred to a 1.2 mL GC sample bottle. The final volume was adjusted to approximately 1 mL under a nitrogen stream. Then, 100 ng of each of the internal standards (2-fluoro-1,10-biphenyl and *p*-terphenyl-d_14_ for PAHs, 4,4′-dichlorobiphenyl for OCPs, pentachloronitrobenzene for PCBs, and C^13^-PCB-208 for PBDEs) was added to the GC bottle. Thereafter, the bottle was tightly sealed up with Teflon-lined butyl rubber septa and aluminum cap. The PAHs, OCPs, PCBs and PBDEs were, respectively, measured with GC/MS (Agilent GC6890/5973MSD, USA), GC/ECD (Agilent GC7890A, ^63^Ni-ECD, USA), GC/MS/MS (Varian 320-MS, USA), and GC/NCI/MS (Agilent 7890A/5975C, USA). More details about the measurements can be seen in [17–19]. 

For quality analysis and quality control, reagent and procedure blanks were included in the measurements. Concentrations of the studied POPs in the blanks were below the detection limits, and recoveries of the objective compounds in the blanks spiked with the standards ranged from 60% to 128%. For all the sediment samples, the recoveries of the surrogates were 59% ~ 94% for PAHs, 75% ~ 102% for OCPs, 76% ~ 103% for PCBs, and 57% ~ 119% for PBDEs. Two replicates were analyzed for some samples and the relative deviation between the replicates was below 30% for the studied POPs. 

The reagents acetone, n-hexane, and dichloromethane (analytical grade, Beijing Reagent, China) were purified by distillation before use. Silica gel (100–200 mesh, Qingdao Marine Chemical, China) was baked at 450°C for 4 h and activated at 130°C for 16 h prior to use. The surrogate standards, internal standards, and working standards (guarantee grade) were purchased from J&K Chemical, USA.

### 2.4. Ecological Risk Assessment of the POPs in the Sediments

Based on the toxicity data of the benthic organisms, the threshold and probable effect concentrations (TEC and PEC) derived from consensus-based sediment quality guidelines for the analytes were used to assess the ecological risks of PAHs, OCPs, and PCBs in the studied sediments [20, 21]. TEC values represent concentrations below which adverse effects to these organisms are not likely, whereas PEC values represent concentrations above which adverse effects are likely. At levels between the TEC and PEC benchmarks, incremental increases in toxicity of sediments have been noted [20]. Two hazard quotients, one representing the threshold effect concentration (TEC-HQ) and the other representing the probable effect concentration (PEC-HQ), were calculated as the ratio of the level of each POP in the sediments to either the TEC or PEC for that POP. When TEC-HQ values were less than 1, rare adverse ecological effects were expected. When TEC-HQ values were greater than 1 but PEC-HQ values were less than 1, adverse ecological effects were possible but less frequent than those observed at the PEC level. Finally, when PEC-HQ values were greater than 1, frequent adverse ecological effects were expected.

Based on the toxicity data of benthic organisms [22], the multiple-species no-observed-effect concentrations (MS NOEC) were derived to assess the ecological risks of PBDEs in the studied sediments [23]. The hazardous quotient (HQ) calculated as the ratio of the measured level to the MS NOEC was used to assess the ecological risk of each PBDE in the sediment. When HQ values were less than 1, rare adverse ecological effects were expected. When HQ values were greater than 1, frequent adverse ecological effects were expected. 

## 3. Results and Discussion

### 3.1. Physicochemical Properties of the Sediments

Physicochemical properties of the 10 sediment samples were measured and shown in Table 1. Overall, the concentrations of available-N in the sediments collected from waste discharging rivers (b2, b3, b5, b6, b8, and b10) were greater than those collected from normal water bodies (b1, b4, b7, and b9), while no much difference between the normal water bodies and the waste discharging rivers was observed for the other parameters. The highest TOC value was detected in b1 (mouth of Hai River), followed by b3 (mouth of Dagu waste discharging river). The physicochemical properties may influence the behavior of POPs in the sediments. 

### 3.2. Concentration Levels of POPs in the Sediments

#### 3.2.1. Concentrations of PAHs in the Sediments

The 16 priority PAHs were all detected in the 10 sediment samples, as shown in Table 2. Overall, the concentration levels of PAHs in the river mouth (b1 and b3) were higher than those in the other locations, the levels of PAHs in the waste discharging rivers were higher than those in the normal water bodies, and the levels of low molecular weight (LMW, 2 ~ 3 rings) PAHs were higher than those of high molecular weight (HMW, 4 ~ 6 rings) PAHs. The ΣPAH_16_ ranged from 274.06 *μ*g/kg to 2656.64 *μ*g/kg, with the mean value being 1198.51 *μ*g/kg. The levels of PAHs in Tianjin coastal area were lower than those in the Pearl River Delta and the Rizhao offshore area but higher than those in Xiamen Bay, Dalian Bay, and the mouth of Yangtze River as well its neighboring sea area in China [24–28]. 

It is generally believed that PAHs of different sources have different structures and compositions, and therefore characteristic ratios of some PAHs could be used to characterize the sources [29–31]. In this study, four ratios, that is, and fluoranthene to pyrene (FLA/PYR), pyrene to benzo(a)pyrene (PYR/BaP), indeno(1,2,3-cd)pyrene to the sum of indeno(1,2,3-cd)pyrene and benzo(ghi)perylene (IcdP/(IcdP+BghiP)), and fluoranthene to the sum of fluoranthene and pyrene (FLA/(FLA+PYR)), were calculated to analyze the sources of PAHs in Tianjin coastal area. The results showed that combustion of fossil fuels (such as coal and gasoline) was the major source of PAHs in the sediments, and in a few places there were inputs of petroleum products. These observations were in agreement with the results obtained in other studies [32, 33].

**Figure 2:**
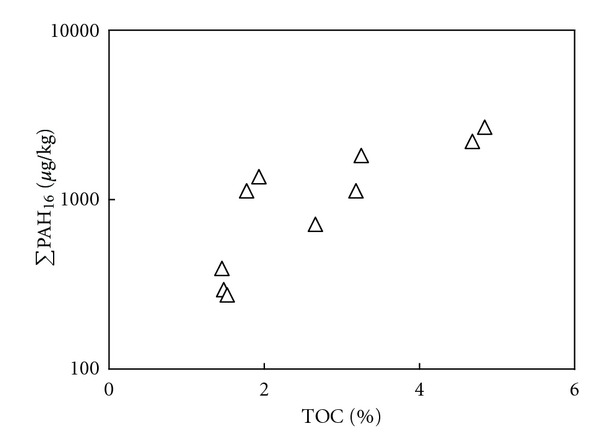
Relationship between TOC and total PAHs in the studied sediments.

It was found that TOC was positively correlated with ΣPAH_16_ (*R* = 0.900,  *P* = 0.000) in this study, as shown in Figure 2. This further demonstrated that the sources of PAHs were nonpoint. PAHs from the sources might enter the water bodies through atmospheric sedimentation, surface runoff, and so forth and accumulated in the sediments owing to the adsorption by the organic matters in the sediments. In addition, the sediments (particularly the polluted ones) were generally in anaerobic conditions under which the biodegradation of PAHs were slow. Therefore, there was good correlation between TOC and ΣPAH_16_.

#### 3.2.2. Concentration Levels of OCPs, PCBs, and PBDEs in the Sediments

**Figure 3:**
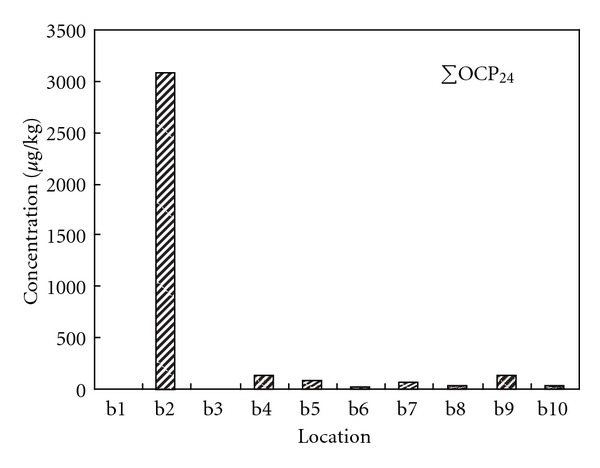
Concentrations of total OCPs in the studied sediments.

**Figure 4:**
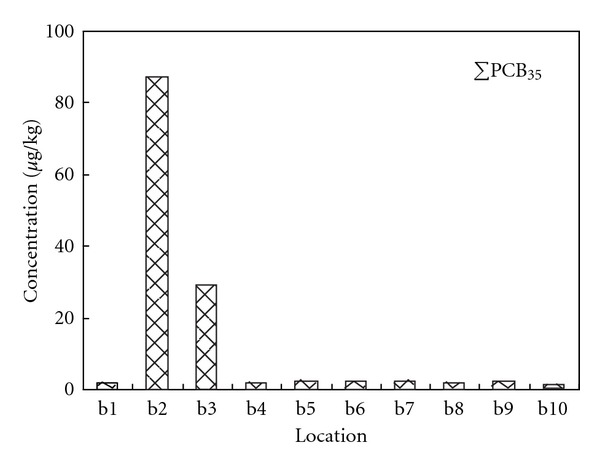
Concentrations of total PCBs in the studied sediments.

**Figure 5:**
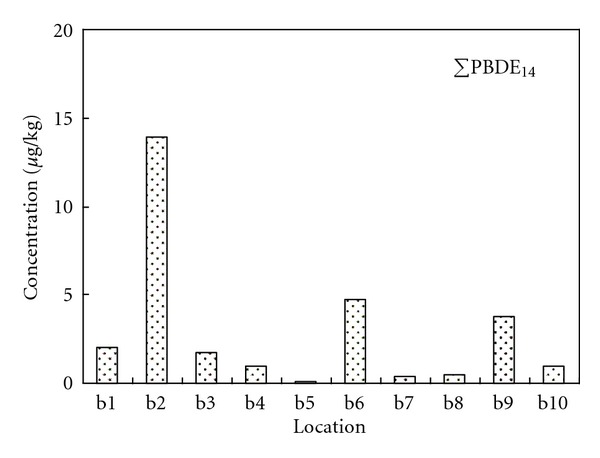
Concentrations of total PBDEs in the studied sediments.

The concentrations of OCPs, PCBs, and PBDEs were generally low in the studied sediments except in the Dagu waste discharging river. Figures 3, 4, and 5 show the total concentrations of 24 OCPs (ΣOCP_24_), 35 PCBs (ΣPCB_35_), and 14 PBDEs (ΣPBDE_14_) in the sediments of various locations. The OCPs data for b1 and b3 were missing due to sample damages. In the Dagu waste discharging river (b2), high concentrations for the halogenated POPs were observed, where hexachlorobenzene (HCB), *α*-benzene hexachloride (*α*-BHC) and *β*-benzene hexachloride (*β*-BHC) were the major OCPs with the concentrations being 1994.99 *μ*g/kg, 337.27 *μ*g/kg and 557.26 *μ*g/kg, respectively, PCB-209, PCB-87, and PCB-70 were the major PCBs with the concentrations being 26.77 *μ*g/kg, 32.30 *μ*g/kg and 16.39 *μ*g/kg, respectively, and BDE-209 was the major PBDE with the concentration being 12.97 *μ*g/kg. The Dagu waste discharging river is a major waste discharging river in Tianjin, with daily discharge amount being over 800 thousand tons [34]. There are several chemical plants such as Tianjin chemical plant, Tianjin Dagu chemical plant located along the banks of the Dagu waste discharging river. These plants historically produced substantial OCPs like HCB, BHCs, and heptachlor epoxide. Wastes from the chemical plants may also contain PCBs and PBDEs that are used in the industry. In the mouth of the Dagu waste discharging river, the concentrations of PCBs and PBDEs were decreased, which were probably due to dilution by the sea water. Tables S1 to S3 in the supplemental materials show the individual concentrations of the OCPs, PCBs, and PBDEs in the studied sediments. 

There was no significant correlation between TOC values and the concentrations of halogenated POPs in the sediment (*R* < 0.400, *P* > 0.300), indicating the sources of the halogenated POPs were of point. Compared to other coastal areas in China, the levels of OCPs in Tianjin were relatively high, exceeding the OCPs in the Pearl River Delta and its adjacent coastal areas of the South Sea, Yangtze River, the offshore of East Sea, and so forth [35–38]. The levels of PCBs and PBDEs were lower than most of the coastal areas in China [39–41].

### 3.3. Ecological Risks of the POPs in the Sediments

The ecological risks of PAHs, OCPs, PCBs, and PBDEs in the studied sediments were assessed on basis of the toxicity data of benthic organisms. Relatively high risks were observed for PAHs, particularly LMW-PAHs. At each sampling location, at least one LMW-PAH had concentration over its TEC, and in several locations (b1, b2, b3, and b10), the concentrations of naphthalene and/or acenaphthene exceeded their PEC values, indicating adverse ecological effects to benthic organisms. Tables 3 and 4, respectively, show the TEC-HQ and PEC-HQ of PAHs in the studied sediments. 

At six locations (b2, b5, b7, b8, b9, and b10), the concentrations of heptachlor epoxide exceeded the TEC value. In the Dagu waste discharging river (b2), the concentrations of heptachlor epoxide and *γ*-BHC exceeded their PEC values (the PEC-HQ of *γ*-BHC was up to 7.49). In the other locations, there were rare risks for OCPs. For PCBs, slight risk was observed in the Dagu waste discharging river (b2) where the ΣPCB_35_ exceeded the TEC but was far less than the PEC; no risk was observed in the other locations. As for PBDEs, all the HQ values were far less than 1 indicating no risk. 

## 4. Conclusions

In the 10 sediments sampled from different surface water bodies in Tianjin coastal area, the 16 priority PAHs were all detected and the mean value of ΣPAH_16_ was 1198.51 *μ*g/kg. The major source of PAHs was the combustion of fossil fuels, with inputs of petroleum products in a few places. The concentration levels of OCPs, PCBs, and PBDEs were generally low except in the Dagu waste discharging river where there were many chemical plants along the banks. The TOC in the sediments had good correlation with ΣPAH_16_ but not with the halogenated POPs. 

There were relatively high risks for PAHs in the studied sediments. At each sampling location, at least one LMW-PAH had concentration over its TEC, and in several locations, the concentrations of naphthalene and/or acenaphthene exceeded their PEC values. In the Dagu waste discharging river, relatively high risk for OCPs and slight risk for PCBs were observed. In the other locations, the risks for the halogenated POPs were rare. 

## Supplementary Material

Source analysis of PAHs in Tianjin coastal area.

## Figures and Tables

**Table 1:** Physicochemical properties of the sampled sediments.

Label	Location	pH	Available-N (mg/L)	Available-P (mg/L)	TOC (%)
b1	Mouth of Hai River	6.1	88.55	24.12	4.83
b2	Dagu waste discharge river	8.3	326.48	16.49	3.18
b3	Mouth of Dagu waste discharge river	7.1	104.34	19.11	4.69
b4	Offshore river	7.5	58.91	<0.10	2.65
b5	Du waste discharging river	8.1	104.72	30.97	1.47
b6	Bei waste discharging river	7.6	75.85	27.65	3.25
b7	Offshore sea	6.9	26.57	25.96	1.45
b8	Yinghe reservoir	7.0	41.97	6.26	1.78
b9	Mouth of Yongding new river	8.1	58.14	37.01	1.53
b10	Beitang waste discharging river	6.9	59.68	7.78	1.94

**Table 2:** Concentrations of PAHs in the sampled sediments (*μ*g/kg).

PAH	b1	b2	b3	b4	b5	b6	b7	b8	b9	b10
Naphthalene	771.09	657.56	582.06	126.68	90.56	145.79	75.69	85.16	76.97	226.21
Acenaphthylene	41.77	15.16	24.72	10.66	4.19	7.83	4.08	42.83	3.91	13.99
Acenaphthene	344.24	77.51	51.62	51.00	22.86	33.48	33.98	21.09	27.04	358.71
Fluorene	245.17	79.40	129.82	208.65	27.88	81.69	40.79	37.79	28.04	255.55
Phenanthrene	289.16	103.49	312.03	54.23	37.76	215.01	56.15	83.57	31.26	134.74
Anthracene	52.72	15.59	60.03	10.54	4.86	41.23	9.69	29.46	6.08	27.11
Fluoranthene	6.72	68.19	256.16	93.34	32.67	275.56	55.69	326.82	32.44	2.41
Pyrene	239.06	55.64	272.13	77.43	24.52	220.19	46.16	194.15	27.22	78.34
Benzo(a)anthracene	71.86	7.18	42.41	15.08	3.66	75.73	3.35	34.37	3.04	30.47
Chrysene	77.46	17.62	117.84	13.90	7.22	171.90	12.73	84.18	7.56	36.50
Benzo(b)fluoranthene	194.85	16.85	83.22	25.53	15.83	106.85	21.32	84.79	13.95	86.15
Benzo(k)fluoranthene	42.01	0.02	52.08	16.11	9.91	66.88	13.34	53.07	8.73	20.19
Benzo(a)pyrene	90.40	6.72	105.78	6.29	3.82	136.30	7.44	30.10	4.27	36.76
Indeno(1,2,3-cd)pyrene	78.22	2.23	59.36	6.37	2.65	130.04	6.30	17.85	2.89	38.89
Dibenzo(a,h)anthracene	22.09	0.51	14.32	1.33	0.54	5.64	1.21	2.98	0.65	9.57
Benzo(ghi)perylene	89.83	2.71	52.93	4.84	2.72	94.66	4.97	11.19	0.01	1.28

∑PAH_16_	2656.64	1126.39	2216.51	721.98	291.63	1808.79	392.88	1139.40	274.06	1356.86

**Table 3:** Threshold effect concentration hazard quotients (TEC-HQ) for PAHs*.

PAH	TEC (*μ*g/kg)	b1	b2	b3	b4	b5	b6	b7	b8	b9	b10
Naphthalene	176	**4.38**	**3.74**	**3.31**	0.72	0.51	0.83	0.43	0.48	0.44	**1.29**
Acenaphthylene	5.87	**7.12**	**2.58**	**4.21**	**1.82**	0.71	**1.33**	0.69	**7.30**	0.67	**2.38**
Acenaphthene	6.71	**51.30**	**11.55**	**7.69**	**7.60**	**3.41**	**4.99**	**5.06**	**3.14**	**4.03**	**53.46**
Fluorene	77.4	**3.17**	**1.03**	**1.68**	**2.70**	0.36	**1.06**	0.53	0.49	0.36	**3.30**
Phenanthrene	204	**1.42**	0.51	**1.53**	0.27	0.19	**1.05**	0.28	0.41	0.15	0.66
Anthracene	57.2	0.92	0.27	**1.05**	0.18	0.08	0.72	0.17	0.51	0.11	0.47
Fluoranthene	423	0.02	0.16	0.61	0.22	0.08	0.65	0.13	0.77	0.08	0.01
Pyrene	195	**1.23**	0.29	**1.40**	0.40	0.13	**1.13**	0.24	**1.00**	0.14	0.40
Benzo(a)anthracene	108	0.67	0.07	0.39	0.14	0.03	0.70	0.03	0.32	0.03	0.28
Chrysene	166	0.47	0.11	0.71	0.08	0.04	**1.04**	0.08	0.51	0.05	0.22
Benzo(b)fluoranthene	240	0.18	0.00	0.22	0.07	0.04	0.28	0.06	0.22	0.04	0.08
Benzo(k)fluoranthene	150	0.60	0.04	0.71	0.04	0.03	0.91	0.05	0.20	0.03	0.25
Benzo(a)pyrene	200	0.39	0.01	0.30	0.03	0.01	0.65	0.03	0.09	0.01	0.19
Indeno(1,2,3-cd)pyrene	33	0.67	0.02	0.43	0.04	0.02	0.17	0.04	0.09	0.02	0.29
Dibenzo(a,h)anthracene	170	0.53	0.02	0.31	0.03	0.02	0.56	0.03	0.07	0.00	0.01

∑PAH_16_	1610	**1.65**	0.70	**1.38**	0.45	0.18	**1.12**	0.24	0.71	0.17	0.84

*Numbers in bold indicate TEC-HQ > 1.

**Table 4:** Possible effect concentration hazard quotients (PEC-HQ) for PAHs*.

PAH	PEC (*μ*g/kg)	b1	b2	b3	b4	b5	b6	b7	b8	b9	b10
Naphthalene	561	**1.37**	**1.17**	**1.04**	0.23	0.16	0.26	0.13	0.15	0.14	0.40
Acenaphthylene	128	0.33	0.12	0.19	0.08	0.03	0.06	0.03	0.33	0.03	0.11
Acenaphthene	88.9	**3.87**	0.87	0.58	0.57	0.26	0.38	0.38	0.24	0.30	**4.03**
Fluorene	536	0.46	0.15	0.24	0.39	0.05	0.15	0.08	0.07	0.05	0.48
Phenanthrene	1170	0.25	0.09	0.27	0.05	0.03	0.18	0.05	0.07	0.03	0.12
Anthracene	845	0.06	0.02	0.07	0.01	0.01	0.05	0.01	0.03	0.01	0.03
Fluoranthene	2230	0.00	0.03	0.11	0.04	0.01	0.12	0.02	0.15	0.01	0.00
Pyrene	1520	0.16	0.04	0.18	0.05	0.02	0.14	0.03	0.13	0.02	0.05
Benzo(a)anthracene	1050	0.07	0.01	0.04	0.01	0.00	0.07	0.00	0.03	0.00	0.03
Chrysene	1290	0.06	0.01	0.09	0.01	0.01	0.13	0.01	0.07	0.01	0.03
Benzo(b)fluoranthene	13400	0.00	0.00	0.00	0.00	0.00	0.00	0.00	0.00	0.00	0.00
Benzo(k)fluoranthene	1450	0.06	0.00	0.07	0.00	0.00	0.09	0.01	0.02	0.00	0.03
Benzo(a)pyrene	3200	0.02	0.00	0.02	0.00	0.00	0.04	0.00	0.01	0.00	0.01
Indeno(1,2,3-cd)pyrene	135	0.16	0.00	0.11	0.01	0.00	0.04	0.01	0.02	0.00	0.07
Dibenzo(a,h)anthracene	3200	0.03	0.00	0.02	0.00	0.00	0.03	0.00	0.00	0.00	0.00

∑PAH_16_	22800	0.12	0.05	0.10	0.03	0.01	0.08	0.02	0.05	0.01	0.06

*Numbers in bold indicate PEC-HQ > 1.
