# Supplementary material for: Concentration Levels and Ecological Risks of Persistent Organic Pollutants in the Surface Sediments of Tianjin Coastal Area, China
Source: ScientificWorldJournal. 2013 Jan 16;2013:417435. doi: 10.1155/2013/417435 (PMC3562668; doi:10.1155/2013/417435)
Supplement: Supplementary file 1 — Source analysis of PAHs in Tianjin coastal area. [file 417435.f1.doc]

**Supplemental Materials**

*Source analysis of PAHs in Tianjin coastal area*

In this study, four ratios were used to characterize the sources, i.e., fluoranthene to pyrene (FLA/PYR), pyrene to benzo(a)pyrene (PYR/BaP), indeno(1,2,3-cd)pyrene to the sum of indeno(1,2,3-cd)pyrene and benzo(ghi)perylene (IcdP/(IcdP+BghiP), and fluoranthene to the sum of fluoranthene and pyrene (FLA/(FLA+PYR)). In general, FLA/PYR>1 indicated the source mainly came from the combustion of fossil fuels, while FLA/PYR<1 indicated the source mainly came from the products of petroleum. PYR/BaP was usually used to differentiate gasoline combustion and coal combustion; PYR/BaP<1 indicated coal combustion, while 1< PYR/BaP<6 indicated gasoline combustion (exhaust emissions). IcdP/(IcdP+BghiP) ≥0.5 indicated the source came from the combustion of grass, wood and coal, 0.4<IcdP/(IcdP+BghiP) <0.5 indicated the source mainly came from the combustion of petroleum type substances, and IcdP/(IcdP+BghiP)<0.4 indicated the source was petroleum. FLA/(FLA+PYR)>0.5 indicated the source was combustion of coal, and FLA/(FLA+PYR) ≤0.5 indicated the source was combustion of petroleum. Figure S1 shows the relationships of the PAH ratios in the studied sediments.

In most of the studied sediments (b2, b4, b5, b6, b7, b8 and b9), FLA/PYR and PYR/BaP were both greater than 1, indicating the source of PAHs mainly came from the combustion of fossil fuels; in b4, b6, b7, b8 and b9, the combustion of coal was the major source because FLA/(FLA+PYR) and IcdP/(IcdP+BghiP) were both greater than 0.5; in b2 and b5, FLA/(FLA+PYR) was greater than 0.5 while whereas IcdP/(IcdP+BghiP) ≤0. 5, indicating both the combustion of coal and the combustion of petroleum were the major sources. In three locations (b1, b3 and b10), FLA/PYR was less than 1 while PYR/BaP was greater than 1, indicating there were input of petroleum products (such as discharge of oily wastewater) and combustion of petroleum. In b1 and b10, FLA/(FLA+PYR) was less than 0.4, further indicating input of petroleum products was a major source. In b2, FLA/(FLA+PYR) was 0.48, indicating the major source was combustion of petroleum type substances. Overall, combustion of fossil fuels (such as coal and gasline) was the major source of PAHs in the studied sediments, and in a few places there was input of petroleum products.

**Figure S1. Relationships of the PAH ratios in the studied sediments**

**Table S1. Concentrations of OCPs in the studied sediments (μg/kg)***

| OCP | b1 | b2 | b3 | b4 | b5 | b6 | b7 | b8 | b9 | b10 |
| --- | --- | --- | --- | --- | --- | --- | --- | --- | --- | --- |
| α-HCH | NA | 337.27 | NA | 2.13 | 3.32 | 4.53 | 1.43 | 0.45 | 4.74 | 0.14 |
| HCB | NA | 1994.99 | NA | 3.74 | 69.96 | 9.98 | 35.68 | 3.21 | 97.99 | 11.52 |
| β-HCH | NA | 557.26 | NA | 23.49 | ND | ND | 3.45 | 0.50 | 3.45 | 8.70 |
| γ- HCH | NA | 88.54 | NA | ND | ND | ND | 0.43 | 0.32 | 0.73 | ND |
| δ- HCH | NA | 32.05 | NA | ND | ND | ND | 0.92 | 0.46 | 1.11 | ND |
| heptachlor | NA | 6.18 | NA | 94.22 | ND | ND | 4.01 | 3.98 | 7.86 | 2.77 |
| aldrin | NA | 11.22 | NA | ND | ND | ND | 1.00 | 0.95 | 0.16 | 2.02 |
| heptachlor epoxide | NA | 52.11 | NA | 0.99 | 4.89 | ND | 4.54 | 5.86 | 5.59 | 2.60 |
| Trans-chlordane | NA | 0.73 | NA | 0.01 | 0.16 | ND | ND | 1.13 | ND | 0.07 |
| o,p'-DDE | NA | 0.29 | NA | 0.05 | 0.54 | 0.45 | ND | 0.51 | ND | 0.32 |
| endosulfan I | NA | 0.85 | NA | 0.02 | ND | 0.02 | ND | 0.45 | ND | 0.09 |
| cis-chlordane | NA | 0.07 | NA | 0.01 | ND | ND | ND | 0.04 | ND | 0.07 |
| dieldrin | NA | 1.05 | NA | 0.01 | 0.11 | 0.06 | ND | 0.77 | ND | 0.07 |
| p,p'-DDE | NA | 1.36 | NA | 0.11 | 0.15 | 0.07 | ND | ND | 0.13 | 0.42 |
| op-ddd | NA | ND | NA | 0.06 | ND | 0.15 | 0.52 | 2.14 | 0.09 | 0.36 |
| endrin | NA | 3.99 | NA | 0.01 | 0.11 | 0.05 | ND | 0.28 | ND | 0.06 |
| endosulfan II | NA | 0.26 | NA | 0.04 | 0.06 | 0.08 | ND | 0.43 | 0.93 | 0.07 |
| p,p'-DDD | NA | 0.11 | NA | 0.09 | 0.19 | 0.19 | 3.19 | 0.43 | 0.72 | 0.65 |
| o,p'-DDT | NA | 0.41 | NA | ND | ND | ND | ND | ND | ND | ND |
| endrin aldehyde | NA | 1.36 | NA | ND | 0.38 | ND | ND | 0.16 | ND | ND |
| endosulfate | NA | ND | NA | ND | ND | ND | ND | ND | ND | ND |
| p,p'-DDT | NA | 1.75 | NA | 0.21 | 0.49 | 0.47 | ND | 1.59 | 0.08 | 0.67 |
| kelthane | NA | 1.98 | NA | 0.13 | 0.38 | 0.02 | ND | 0.55 | ND | 0.12 |
| mirxe | NA | 9.52 | NA | 1.52 | 3.92 | 0.60 | 4.32 | 4.04 | 4.29 | 3.56 |
| ΣOCP_24_ | NA | 3103.36 | NA | 126.82 | 84.65 | 16.68 | 59.50 | 28.24 | 127.88 | 34.27 |

***** NA indicates the samples were not analyzed; ND indicates the samples were not detected (below detection limits)

**Table S2. Concentrations of PCBs in the studied sediments (μg/kg)***

| PCB | b1 | b2 | b3 | b4 | b5 | b6 | b7 | b8 | b9 | b10 |
| --- | --- | --- | --- | --- | --- | --- | --- | --- | --- | --- |
| PCB-18 | ND | ND | 0.71 | ND | ND | ND | ND | ND | ND | ND |
| PCB-44 | ND | ND | ND | ND | ND | ND | ND | ND | ND | ND |
| PCB-49 | ND | ND | ND | ND | ND | ND | ND | ND | ND | ND |
| PCB-52 | ND | ND | ND | ND | ND | ND | ND | ND | ND | ND |
| PCB-37 | ND | ND | ND | ND | ND | ND | ND | ND | ND | ND |
| PCB-70 | ND | 16.39 | 14.49 | ND | ND | ND | ND | ND | ND | ND |
| PCB-74 | ND | ND | 8.20 | ND | ND | ND | ND | ND | ND | ND |
| PCB-87 | ND | 32.30 | 0.21 | ND | ND | ND | ND | ND | ND | ND |
| PCB-99 | ND | 0.85 | ND | ND | ND | ND | ND | ND | ND | ND |
| PCB-114 | ND | 0.12 | ND | ND | ND | ND | ND | ND | ND | ND |
| PCB-81 | ND | 0.15 | 0.02 | ND | ND | ND | ND | ND | ND | ND |
| PCB-101 | ND | 2.63 | 0.50 | ND | ND | 0.41 | ND | ND | ND | ND |
| PCB-77 | ND | ND | 0.09 | ND | ND | ND | ND | ND | ND | ND |
| PCB-151 | ND | 4.38 | 1.03 | ND | ND | ND | ND | ND | ND | ND |
| PCB-119 | ND | 0.28 | 0.04 | ND | ND | 0.02 | ND | ND | ND | ND |
| PCB-105 | ND | 0.16 | 0.01 | ND | ND | 0.01 | ND | ND | ND | ND |
| PCB-123 | ND | 0.06 | 0.02 | ND | ND | ND | ND | ND | ND | ND |
| PCB-153 | ND | 0.39 | 0.01 | ND | ND | ND | ND | ND | ND | ND |
| PCB-157 | ND | 0.39 | 0.01 | ND | ND | 0.02 | ND | ND | ND | ND |
| PCB-128 | ND | 0.39 | 0.24 | ND | 0.12 | 0.01 | ND | ND | ND | ND |
| PCB-158 | ND | 0.04 | 0.01 | ND | 0.02 | ND | ND | ND | ND | ND |
| PCB-126 | ND | 0.02 | 0.01 | ND | ND | ND | ND | ND | ND | ND |
| PCB-177 | ND | 0.19 | ND | ND | ND | ND | ND | ND | ND | ND |
| PCB-180 | ND | 0.18 | 0.05 | ND | ND | ND | ND | ND | ND | ND |
| PCB-138 | ND | 0.01 | ND | ND | ND | 0.01 | ND | ND | ND | ND |
| PCB-187 | ND | 0.12 | 0.03 | ND | ND | 0.01 | ND | ND | ND | ND |
| PCB-156 | ND | 0.06 | 0.02 | ND | ND | ND | ND | ND | ND | ND |
| PCB-168 | ND | 0.11 | 0.03 | ND | ND | ND | ND | ND | ND | ND |
| PCB-183 | ND | 0.11 | 0.03 | 0.01 | ND | 0.03 | ND | ND | 0.01 | ND |
| PCB-169 | ND | 0.00 | 0.03 | ND | ND | ND | ND | ND | ND | ND |
| PCB-170 | ND | 0.05 | 0.01 | ND | ND | 0.01 | ND | ND | ND | ND |
| PCB-199 | ND | 0.99 | 0.26 | ND | ND | 0.02 | ND | ND | ND | ND |
| PCB-189 | ND | 0.10 | 0.03 | ND | ND | 0.01 | ND | ND | ND | ND |
| PCB-194 | ND | 0.09 | 0.03 | 0.01 | 0.01 | 0.02 | ND | ND | 0.01 | ND |
| PCB-209 | 1.98 | 26.77 | 3.15 | 1.98 | 2.12 | 1.90 | 2.38 | 2.06 | 2.56 | 1.22 |
| ΣPCB_35_ | 1.98 | 87.31 | 29.25 | 1.99 | 2.28 | 2.47 | 2.38 | 2.06 | 2.57 | 1.22 |

***** ND indicates the samples were not detected (below detection limits)

**Table S3. Concentrations of PBDEs in the studied sediments (μg/kg**)*

| PBDE | b1 | b2 | b3 | b4 | b5 | b6 | b7 | b8 | b9 | b10 |
| --- | --- | --- | --- | --- | --- | --- | --- | --- | --- | --- |
| PBDE-17 | 0.02 | 0.01 | ND | 0.19 | ND | 0.01 | ND | 0.08 | 0.55 | 0.01 |
| PBDE-28 | 0.65 | 0.47 | 0.37 | ND | ND | 0.39 | 0.33 | 0.36 | 0.01 | 0.24 |
| PBDE-47 | 0.01 | 0.02 | 0.03 | 0.04 | ND | 0.11 | ND | ND | 1.85 | 0.33 |
| PBDE-66 | ND | 0.10 | 0.07 | ND | ND | 0.01 | ND | ND | 0.01 | 0.01 |
| PBDE-71 | 0.03 | ND | ND | 0.14 | ND | 0.01 | ND | ND | 0.06 | 0.15 |
| PBDE-100 | 0.01 | 0.02 | 0.06 | ND | ND | 0.01 | ND | ND | 1.24 | 0.03 |
| PBDE-99 | 0.58 | 0.02 | ND | 0.10 | ND | 0.04 | ND | ND | 0.02 | 0.12 |
| PBDE-85 | 0.20 | 0.01 | 0.21 | 0.11 | ND | 2.12 | ND | ND | 0.01 | 0.01 |
| PBDE-154 | 0.08 | 0.18 | 0.04 | 0.08 | ND | 0.24 | ND | ND | 0.01 | 0.01 |
| PBDE-153 | 0.01 | 0.02 | 0.18 | 0.17 | ND | 0.09 | ND | ND | 0.01 | 0.02 |
| PBDE-138 | 0.13 | 0.02 | 0.09 | 0.15 | 0.07 | 0.56 | ND | ND | ND | 0.03 |
| PBDE-183 | 0.04 | 0.01 | 0.21 | ND | ND | 0.58 | 0.01 | ND | 0.02 | 0.02 |
| PBDE-190 | 0.01 | 0.02 | 0.51 | ND | ND | 0.32 | ND | ND | ND | 0.01 |
| PBDE-209 | 0.25 | 12.97 | ND | ND | ND | 0.23 | ND | ND | ND | 0.00 |
| ΣPBDE_14_ | 2.03 | 13.88 | 1.77 | 1.01 | 0.12 | 4.73 | 0.39 | 0.49 | 3.79 | 1.01 |

***** ND indicates the samples were not detected (below detection limits)
